# New trends in nanobiotechnology

**DOI:** 10.3762/bjnano.14.32

**Published:** 2023-03-27

**Authors:** Pau-Loke Show, Kit Wayne Chew, Wee-Jun Ong, Sunita Varjani, Joon Ching Juan

**Affiliations:** 1 Department of Chemical Engineering, Khalifa University, P.O. Box 127788, Abu Dhabi, United Arab Emirates; 10 Nanotechnology & Catalysis Research Centre (NanoCat), Institute of Postgraduate Studies, University Malaya, 50603, Kuala Lumpur, Malaysia; 2 Zhejiang Provincial Key Laboratory for Subtropical Water Environment and Marine Biological Resources Protection, Wenzhou University, Wenzhou, 325035, China; 3 Department of Sustainable Engineering, Saveetha School of Engineering, SIMATS, Chennai, 602105, India; 4 Department of Chemical and Environmental Engineering, Faculty of Science and Engineering, University of Nottingham Malaysia, Jalan Broga, 43500, Semenyih, Selangor Darul Ehsan, Malaysia; 5 School of Chemistry, Chemical Engineering and Biotechnology, Nanyang Technological University, 62 Nanyang Drive, Singapore, 637459 Singapore; 6 School of Energy and Chemical Engineering, Xiamen University Malaysia, Selangor Darul Ehsan, Selangor, 43900, Malaysia; 7 Center of Excellence for NaNo Energy & Catalysis Technology (CONNECT), Xiamen University Malaysia, Selangor Darul Ehsan, Selangor, 43900, Malaysia; 8 Centre for Energy and Environmental Sustainability, Lucknow, India; 9 School of Energy and Environment, City University of Hong Kong, Tat Chee Avenue, Kowloon, 999077, Hong Kong

**Keywords:** biocompatible nanoparticles, cancer cells, carrageenan, cytotoxic selectivity, green synthesis methods, nanobiotechnology, SARS-CoV-2, self-assembly, wet chemical reduction

The widespread use of nanotechnology has reached almost every sector in our daily lives and amazed the world by offering various potential applications in these sectors. The uprising wave of nanotechnology and its application are now prominent in the fields of chemistry and biomedicine, which are vital as these fields serve as a basis for the discovery of new molecules that may benefit humans. Nanotechnology contributed to the advancement of promising techniques either by the implementation of existing methods or by the establishment of new ones. Researchers in academia and industry sectors working in areas of biochemistry, chemical engineering, molecular biology, and genetics are likely to come across the advantages of applying nanobiotechnology tools in their studies. This profound technological advantage has brought many research laboratories to globally exchange ideas and promote intensive international scientific collaborations to further increase the level of understanding of applying nanotechnology to biological systems.

This thematic issue aims to provide vital findings to support new research and innovations utilizing recent trends in nanobiotechnological processes to encourage the development of these converging technologies for a sustainable economic growth.

The synthesis and the characterization of nanoscale biomaterials, the innovative applications of “smart nanoparticles”, and the technological/biological impact of nanoscale systems are just some of the areas of focus in the field known as nanobiotechnology [1]. Nanobiotechnology has a wide array of applications: from organ-on-a-chip technologies to nanobiosensors and nanocatalysts for advanced characterisation and imaging tools, from intelligent drug delivery systems to artificial bioconstructs, and from functional nanostructured surfaces to smart materials and nanofluidics. In all these applications, it is important to consider the nanotoxicological and possible harmful impact of nanomaterials on living organisms [2]. In fact, the evaluation of the safety of a novel nanodevice is a process that should start at the very first step of concept and design. Particular attention should also be paid to the translational and regulatory aspects of nanobiomedical devices in order to enable them to be used in future clinical practice [3]. With proper consideration of these impacts, the implementation of nanotechnology tools can then be done in a safe manner.

In this thematic issue we invited many authors to contribute with manuscripts on novel concepts, ingenious designs, and promising applications in the field of nanobiotechnology. The submitted works were expected to feature innovative areas such as nanomaterials applied in biotechnology; nanoparticles used in environmental science and technology; nanosensors used in biosystems; nanomedicine in the context of biochemical engineering; micro- and nanofluidics; micro- and nano-electromechanical systems; nanoscience and nanotoxicology; nanotechnology applied in biology, medicine, food, environmental and agriculture sectors; environmental engineering and chemical engineering; nanoscale electrochemisty in biotechnology; computational nanochemistry in biotechnology; and life cycle assessment of nanobiotechnology.

The works presented in this thematic issue covered topics related to new concepts and ideas pertaining to the design and development of nanobiotechnology. These works include “The role of deep eutectic solvents and carrageenan in synthesizing biocompatible anisotropic metal nanoparticles” [4]. This review sheds light onto significant works involving the synthesis of metal nanoparticles using environmentally friendly wet chemical methods in which carrageenan is the main resource. The review summarises the possibility of creating a safe and non-toxic path to the synthesis of nanomaterials while maintaining its properties, such as morphology, yield and monodispersity. The introduction of a deep eutectic solvent as a cost-effective and green solvent was reviewed, where the usage of these solvents enabled the extraction and formation of desired nanostructures. The work also records the advantages and disadvantages of wet chemical reduction methods which use surfactants, and explores the in vitro and in vivo cytotoxicity of the synthesized anisotropic nanoparticles. A portion of the work looks into the possible integration of nanotechnology in deep eutectic solvent extractions and also the use of carrageenan as a safe stabilizing agent for nanomaterials synthesis. The review is concluded providing an outlook of these two components (i.e., deep eutectic solvents and carrageenan) as alternatives for the formation of plasmonic metal nanoparticles. The importance of applying these tools to improve the physicochemical properties and biocompatibility of the nanomaterials is also discussed.

The thematic issue also recorded a work on the topic of “Self-assembly of amino acids toward functional biomaterials” [5], where the role of biomaterials in nanobiotechnology is discussed. In this review, the latest advances in amino acid self-assembly and properties associated with the process and yielded products are highlighted. The self-assembly methods in focus included single amino acid self-assembly, functional amino acid self-assembly, amino acid and metal ion coordination self-assembly, and amino acid regulatory functional molecule self-assembly. Many works on self-assembly have shown low synthesis cost, ease of modelling, and good biocompatibility of the generated biomolecules. The review discusses the introduction and case studies of different types of self-assembly, applying examples on the application of the method. Finally, the review summarizes the use of nanotechnology in self-assembly methods and the challenges to adapt these nanomaterials to commercial applications.

Some other hot topics in the field of nanobiotechnology were also covered in the thematic issue. One of these topics is on the “Design and selection of peptides to block the SARS-CoV-2 receptor binding domain by molecular docking” [6]. This research work showcases peptides that are capable to bind and neutralize the SARS-CoV-2 virus through molecular docking. The latest developments of the molecular docking of peptides by molecular dynamics were investigated to understand the interaction between peptides with physiological proteins. Through the study, the selection and rapid design of peptides based on peptide binding sites, hydrogen bond number, and binding affinity were obtained. It was also concluded the potential role of these peptides in the prevention of infection caused by SARS-CoV-2. Another important topic covered in this thematic issue is presented in this article: “In search of cytotoxic selectivity on cancer cells with biogenically synthesized Ag/AgCl nanoparticles” [7]. This work explores the use of pineapple waste for the synthesis of silver and silver chloride nanoparticles, along with the analysis of the selective cytotoxicity of these nanoparticles on healthy and cancerous cells. The work aims to contribute to the production of alternative nanomaterials obtained from waste for therapeutic applications with emphasis on disease mitigation. Green synthesis methods were firstly applied for the biosynthesis of silver nanoparticles, along with silver chloride nanoparticles as there were chlorine salts in the pineapple peels which enable the formation of silver chloride. These nanoparticles were then characterized and tested regarding their cytotoxicity activity on cancer and healthy cells. The results showed a selective cytotoxicity of the nanoparticles towards cancer cell compared to that towards monocytes. This finding gives rise to the development of a new system where cytotoxicity can be selective. This may benefit future research in the field of nanoparticle synthesis for medical treatments.

The collection of comprehensive reviews and studies assembled in this thematic issue on nanobiotechnology trends provides useful and new scientific knowledge regarding the advancement of nanobiotechnology for science, technological, and engineering-related applications. A total of five high quality works were published within the thematic issue, with great support from researchers in various continents. The guest editors wish to express their gratitude to all the contributors, authors and reviewers, who have collectively ensured and maintained the standards of scientific quality within the works published. Finally, we also thank Dr. Wendy Patterson, Dr. Lasma Gailite, and Dr. Barbara Hissa for their support in the development of the “New Trends in Nanobiotechnology” thematic issue.
